# Ghrelin and glucagon-like peptide-1 according to body adiposity and glucose homeostasis

**DOI:** 10.20945/2359-3997000000611

**Published:** 2023-05-10

**Authors:** Karynne Grutter Lopes, Vicente Lopes da Silva, Fernanda de Azevedo Marques Lopes, Eliete Bouskela, Maria das Graças Coelho de Souza, Luiz Guilherme Kraemer-Aguiar

**Affiliations:** 1 Universidade do Estado do Rio de Janeiro Hospital Universitário Pedro Ernesto Centro de Pesquisas Clínicas Multiusuário Rio de Janeiro RJ Brasil Unidade de Obesidade, Centro de Pesquisas Clínicas Multiusuário (CePeM), Hospital Universitário Pedro Ernesto (HUPE), Universidade do Estado do Rio de Janeiro, Rio de Janeiro, RJ, Brasil; 2 Universidade do Estado do Rio de Janeiro Faculdade de Ciências Médicas Programa de Pós-graduação em Fisiopatologia Clínica e Experimental Rio de Janeiro RJ Brasil Programa de Pós-graduação em Fisiopatologia Clínica e Experimental (Fisclinex), Faculdade de Ciências Médicas, Universidade do Estado do Rio de Janeiro, Rio de Janeiro, RJ, Brasil; 3 Universidade do Estado do Rio de Janeiro Laboratório de Pesquisa Clínica e Experimental em Biologia Vascular Rio de Janeiro RJ Brasil Laboratório de Pesquisa Clínica e Experimental em Biologia Vascular (BioVasc), Universidade do Estado do Rio de Janeiro, Rio de Janeiro, RJ, Brasil; 4 Universidade do Estado do Rio de Janeiro Faculdade de Ciências Médicas Departamento de Medicina Interna Rio de Janeiro RJ Brasil Endocrinologia, Departamento de Medicina Interna, Faculdade de Ciências Médicas, Universidade do Estado do Rio de Janeiro, Rio de Janeiro, RJ, Brasil

**Keywords:** Obesity, glucose tolerance, inflammation, gastrointestinal peptides

## Abstract

**Objective:**

We investigated the biological behavior of ghrelin and glucagon-like peptide-1 (GLP-1) after a standard liquid meal according to body adiposity and glucose homeostasis.

**Subjects and methods:**

This cross-sectional study included 41 individuals (92.7% women; aged 38.3 ± 7.8 years; BMI 32.2 ± 5.5 kg/m²) allocated into three groups according to body adiposity and glucose homeostasis, as follows: normoglycemic eutrophic controls (CON, n = 11), normoglycemic with obesity (NOB, n = 15), and dysglycemic with obesity (DOB, n = 15). They were tested at fasting and 30 and 60 min after the ingestion of a standard liquid meal in which we measured active ghrelin, active GLP-1, insulin, and plasma glucose levels.

**Results:**

As expected, DOB exhibited the worst metabolic status (glucose, insulin, HOMA-IR, HbA1c) and an inflammatory status (TNF-α) at fasting, besides a more significant increase in glucose than postprandial NOB (p ≤ 0.05). At fasting, no differences between groups were detected in lipid profile, ghrelin, and GLP-1 (p ≥ 0.06). After the standard meal, all groups exhibited a reduction in ghrelin levels between fasting vs. 60 min (p ≤ 0.02). Additionally, we noticed that GLP-1 and insulin increased equally in all groups after the standard meal (fasting vs. 30 and 60 min). Although glucose levels increased in all groups after meal intake, these changes were significantly more significant in DOB vs. CON and NOB at 30 and 60 min post-meal (p ≤ 0.05).

**Conclusions:**

Time course of ghrelin and GLP-1 levels during the postprandial period was not influenced by body adiposity or glucose homeostasis. Similar behaviors occurred in controls and patients with obesity, independently of glucose homeostasis.

## INTRODUCTION

The role of the gut-brain axis in energy homeostasis has been of interest in the past decades and is involved in the pathogenesis and treatment of metabolic disease ( 1 , 2 ). Enteroendocrine cells throughout the gastrointestinal tract (GI) produce several peptides, such as cholecystokinin (CCK), peptide YY, glucose-dependent insulinotropic polypeptide (GIP), ghrelin, and glucagon-like peptide-1 (GLP-1), acting in concert to regulate GI motility, glucose homeostasis, appetite, and food intake ( 3 , 4 ).

Ghrelin, an orexigenic peptide, is secreted primarily from the stomach and duodenum ( 5 ). In normal-weight individuals at fasting status, ghrelin levels are elevated, inducing hunger and preventing hypoglycemia through increased glucagon secretion and decreased glucose-stimulated insulin secretion ( 6 ).

GLP-1, an incretin hormone, is secreted post-meal ingestion from enteroendocrine L cells in the distal jejunum and ileum and from neurons in the brainstem and hypothalamus. GLP-1 stimulates glucose-dependent insulin release from pancreatic islet β-cells, inhibits glucagon secretion, and delays gastric emptying, thereby slowing nutrient absorption. These actions contribute to the reduction of postprandial glycemia and enhanced satiety ( 4 ).

It has been suggested that individuals with obesity and insulin resistance/type 2 diabetes mellitus (T2DM) retain sensitivity to the actions of gut hormones ( 7 ). In general, low fasting levels and attenuated postprandial responses of ghrelin and GLP-1 are observed in individuals with obesity ( 8 , 9 ). However, little is known about the time-course of postprandial levels of ghrelin and GLP-1 and their relationships with adiposity and glucose homeostasis ( 5 , 10 ). We hypothesize that excessive adiposity and metabolic and inflammatory status are independently associated with suppressed ghrelin levels during the postprandial period ( 8 ). Therefore, we compared postprandial responses of ghrelin and GLP-1, insulin, and glucose after a standard liquid meal in normoglycemic eutrophic healthy individuals *vs.* normoglycemic and dysglycemic individuals paired for excessive adiposity.

## SUBJECTS AND METHODS

### Subjects

A total of 88 patients were recruited in our outpatient care unit for patients with obesity. Forty-one patients were included in our study (92.7% women; aged 38.3 ± 7.8 years; body mass index [BMI] 32.6 ± 5.0 kg/m²). They were allocated into three groups according to body adiposity and glucose homeostasis, as follows: a) normoglycemic eutrophic as controls (CON; *n* = 11), b) normoglycemic with obesity (NOB; *n* = 15), and c) dysglycemic with obesity (DOB; *n* = 15). BMI was used to classify normal body mass or those with obesity ( 11 ). Different degrees of glucose tolerance were classified according to the American Diabetes Association (ADA) ( 12 ). Patients with fasting plasma glucose (PG) < 100 mg/dL, or 2 h PG during the oral glucose tolerance test (OGTT; 75 g glucose anhydrous) < 140 mg/dL, were classified as normoglycemic, and those with fasting PG 100-125 mg/dL or 2 h PG > 140 mg/dL were classified as dysglycemic. We also used glycated hemoglobin levels (HbA1c, > 5.7%) as a criterion for dysglycemia. Exclusion criteria were cancer, uncontrolled hypertension, chronic obstructive pulmonary, kidney, liver, or gastrointestinal diseases, unstable dietary history, use of immunosuppressive agents and probiotics in the last 6 months, HIV infection, hepatitis B or C, pregnancy, smoking, and alcoholism. Recruitment, pre-participation screening, and data collection occurred for 1 year.

### Ethical approval

The local research ethics committee approved this cross-sectional study (CAAE: 22032113900005259, approved on October 09, 2013) and registered it on ClinicalTrials.gov (ID number NCT03178006). Participants received a complete explanation of the benefits and risks of the study before providing written informed consent. All procedures were performed according to the principles of the Declaration of Helsinki.

### Experimental design

The pre-participation screening, collection of written informed consent, and data collection occurred between 7-11 am over a period of 2 days, interspersed with at least 24-48-h intervals, in the following order: a) Visit 1: clinical history, physical examination, and anthropometric/hemodynamic measurements, and b) Visit 2: body composition and blood sample collection to assess metabolic and inflammatory biomarkers and gastrointestinal peptides. The participants were instructed to fast for 8 h (for Visit 2), not to practise any physical activity, and to avoid caffeinated beverages for 24 h before the experimental session.

**Figure 1:**
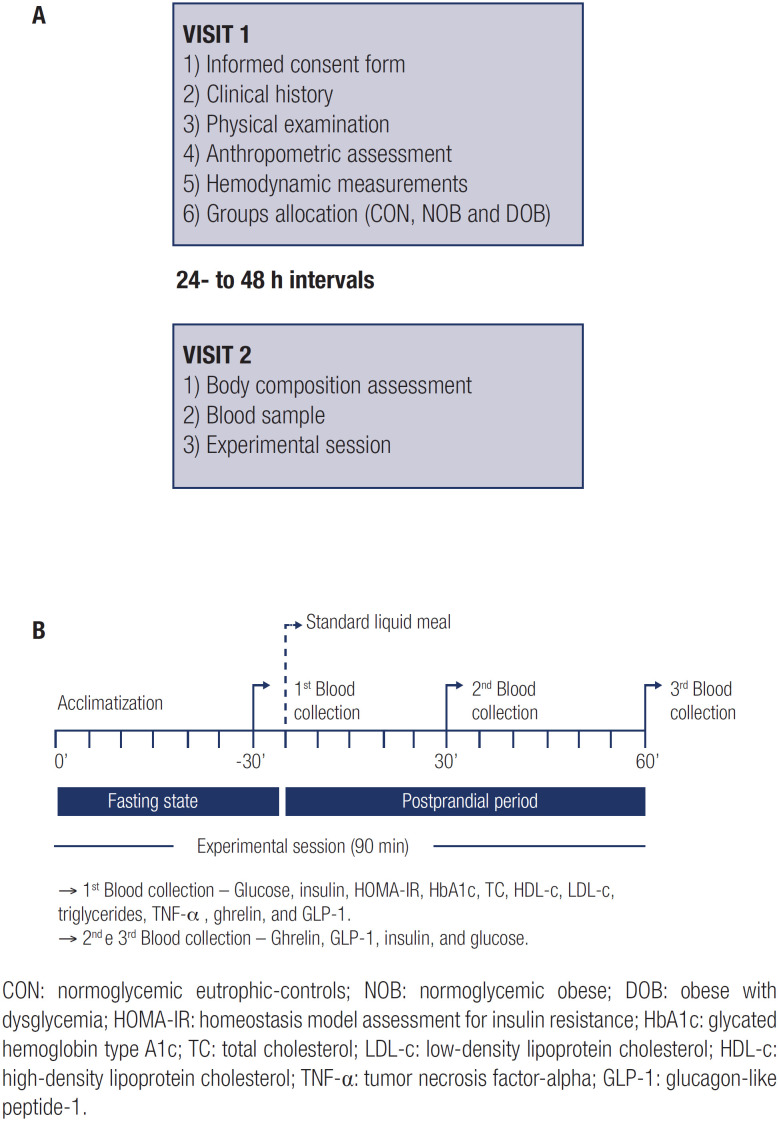
Study design ( **A** ) and experimental session ( **B** ).

After 30 min in a supine position in a quiet and temperature-controlled room (24 ± 1 °C), the experimental protocol was started and involved blood collection in the fasting state and the postprandial period (30 and 60 min post-meal). After the first 5 min, the participants ingested a standard liquid meal, not exceeding 5 min of ingestion time. Previously, an intravenous catheter was inserted in the cubital vein and maintained during the experimental session for collecting blood samples. The standard liquid meal (Nutridrink^®^, Danone^®^, Paris, France; 200 mL) was composed of 300 kcal (49% carbohydrates, 35% lipids, 16% proteins) and was the same for all groups. Figure 1A depicts the study design, and Figure 1B depicts the procedures performed during the experimental session.

## PROCEDURES

### Anthropometry, body composition, and hemodynamic measurements

An electronic scale and stadiometer (Filizola^®^, São Paulo, Brazil) were used to measure body mass and height. BMI was calculated as kg/m^2^. Body circumferences were measured by flexible measuring tape according to standard procedures. Total lean and fat mass were evaluated by a bioimpedance analyzer (Biodynamics 450 Biodynamics Corporation, Shoreline, WA, USA), according to the manufacturer's instructions. Blood pressure was assessed by a semiautomated oscillometric device (G-Tech^TM^ BSP11, Hangzhou, Zhejiang, China) in a controlled and quiet environment, after 10 min of resting in a sitting position, according to standard guidelines ( 13 ).

### Laboratory evaluations

Blood samples were collected through venipuncture into serum, plasma EDTA, and fluoride tubes. In order to prevent the active forms of ghrelin and GLP-1 from degradation, immediately after collection a serine protease inhibitor (Pefabloc SC, Roche Diagnostics GmbH, Mannheim, Germany) and DPP4 inhibitor (EMD Millipore Corporation, MO, USA) were added to plasma EDTA tubes, yielding a 2-mM and 50-νM final concentration of these inhibitors, respectively. Then, plasma EDTA tubes were centrifuged at 1,000 rpm at 4 °C for 10 min, while tubes for plasma fluoride and serum were centrifuged at 3,000 rpm at 18 °C for 10 min. Plasma and serum samples were transferred into cryotubes and stored at −80 °C until analysis.

Plasma levels of TNF-α were assessed exclusively during a fasted state using a commercially available kit (Human TNF-α HS Quantikine ELISA Kit, MN, USA). Blood levels of glycated hemoglobin type A1c (HbA1c) were measured by turbidimetric inhibition using the Automatic Analyzer A25 (BioSystems, Barcelona, Spain).

At baseline (fasting), 30- and 60-min post-meal intake, plasma levels of active ghrelin (also known as n-octanoyl ghrelin), insulin, and active GLP-1 were assessed by Milliplex^®^ MAP Human Metabolic Hormone Magnetic Bead Panel (Merck-Millipore, Billerica, MA, USA) and High Sensitivity GLP-1 Active Chemiluminescent Kit (Merck-Millipore, Billerica, MA, USA), respectively. Additionally, plasma glucose was evaluated in plasma fluoride through the glucose oxidase colorimetric method. Serum levels of triglycerides, total cholesterol, and high-density lipoprotein cholesterol (HDL-c) were assessed by glycerol phosphate oxidase/peroxidase, cholesterol oxidase/peroxidase, and direct colorimetric methods, respectively. These analyses were all performed by commercially available kits appropriate for the Automatic Analyzer A25 (BioSystems, Barcelona, Spain). LDL-c was calculated by the Friedewald equation ( 14 ). The homeostatic model assessment for insulin resistance (HOMA-IR) was used to quantify fasting insulin resistance [(PG in mmol/L × insulin in μIU/mL)/22.5] ( 15 ). All analyses were performed according to the instructions provided by the manufacturers. The intra- and inter-assay coefficients of variation for all analyses were less than 15%.

### Statistical analysis

Statistical power was calculated using the G*Power version 3.1.9.4 software (Universitat of Kiel, Kiel, Germany), considering a sample power of 0.8, α error probability of 0.05, and effect size of 0.5, resulting in a total sample size of 27 subjects. The Shapiro-Wilk test ratified data normality. When appropriate, data were expressed as mean ± standard deviation (SD) or medians [percentiles 25-75]. The chi-square test compared categorical variables. One-way ANOVA was used for comparisons between groups, and 2-way ANOVA with repeated measures (factors: group and time) to test differences between experimental situations, followed by Bonferroni *post hoc* tests in the event of significant *F* ratios. Pearson correlations were calculated to verify the associations between ghrelin, GLP-1, insulin, and glucose changes after standard meal intake. Statistical calculations were performed using GraphPad Prism^®^ software (version 6.0, La Jolla, CA, USA) and NCSS^TM^ statistical software (Kaysville, UT, USA). The statistical significance level was set at *p ≤* 0.05.

## RESULTS

Baseline demographic characteristics, body composition, and laboratory variables of the participants are presented in Table 1 . DOB was older than NOB ( *p =* 0.02). As expected, individuals with hypertension (53.3%) were more prevalent in DOB *vs.* CON and NOB ( *p* = 0.003). The weight, BMI, waist, hip circumference, and lean and fat mass were higher in NOB and DOB *vs.* CON ( *p* ≤ 0.02, for all outcomes). On the other hand, no differences between groups were found for the percentage of women, hip circumference, waist-to-hip ratio, and heart rate ( *p ≥* 0.07, for all outcomes).

**Table 1:** Results of demographic characteristics, body composition, and laboratory variables of the study groups

		CON ( *n* = 11)	NOB ( *n* = 15)	DOB ( *n* = 15)	*p* -value
Demographic characteristics					
	Age (years)	39 ± 7	33 ± 7	41 ± 6†	**0.02**
	Women (n, %)	10 (90.9)	13 (86.7)	15 (100)	0.36
	Weight (kg)	63.4 ± 8.6	95.1 ± 12.2 *	92.2 ± 12.6 *	**<0.001**
	Body mass index (kg/m²)	23.6 ± 1.5	33.8 ± 3.5 *	35.4 ± 2.9 *	**<0.001**
	Waist circumference (cm)	85.4 ± 5.0	110.1 ± 6.8 *	107.4 ± 7.4 *	**<0.001**
	Hip circumference (cm)	97 [92-100]	116 [113-127]	115.5 [110-122]	0.07
	Waist-to-hip ratio	0.84 ± 0.13	0.92 ± 0.05	0.92 ± 0.06	0.07
	Hypertension (n, %)	0 (0)	2 (13.3)	8 (53.3) f	**0.003**
	Heart rate (bpm)	70 ± 8	69 ± 7	66 ± 7	0.29
Body composition					
	Lean mass (kg)	46.2 ± 6.8	56.1 ± 7.9 *	54.8 ± 6.5 *	**0.02**
	Fat mass (%)	26.0 ± 2.7	40.0 ± 4.0 *	40.4 ± 2.2 *	**<0.001**
Biochemical profile					
	Glucose (mg/dL)	95.8 ± 8.1	97.9 ± 7.1	140.3 ± 53.6 * †	**0.009**
	Insulin (pg/mL)	268.0 ± 280.7	313.2 ± 103.9	566.7 ± 477.4 *	**0.05**
	HOMA-IR	1.68 ± 1.75	1.91 ± 0.60	4.51 ± 3.51 * †	**0.005**
	HbA1c (%)	5.3 [5.0-5.4]	5.5 [5.3-5.6]	6.1 [6.0-8.4] *	**<0.001**
	TC (mg/dL)	183.4 ± 33.9	178.7 ± 38.8	175 ± 23.4	0.34
	HDL-c (mg/dL)	51.5 [42-59.7]	54 [40-62.5]	46 [39-49]	0.22
	LDL-c (mg/dL)	106 [93-133]	104 [88-117.5]	111 [93-119]	0.85
	Triglycerides (mg/dL)	96.5 [74.2-131.5]	83 [65-135.5]	118 [86-155]	0.22
	TNF-α (pg/mL)	0.56 ± 0.25	0.76 ± 0.40	0.96 ± 0.41 *	**0.03**
	Ghrelin (pg/mL)	81.6 ± 60.6	73.5 ± 35.5	45.3 ± 24.1	0.06
	GLP-1 (pM)	0.62 [0.43-0.96]	0.59 [0.50-0.73]	0.84 [0.63-0.89]	0.23

CON: normoglycemic eutrophic; NOB: normoglycemic obese; DOB: obese with dysglycemia; HOMA-IR: homeostasis model assessment of insulin resistance; HbA1c: glycated hemoglobin type A1c; TC: total cholesterol; LDL-c: low-density lipoprotein cholesterol; HDL-c: high-density lipoprotein cholesterol; TNF-α: tumor necrosis factor-alpha; GLP-1: glucagon-like peptide-1.

*P* -values of One-way ANOVA or chi-square test; results expressed as mean ± SD, median [percentiles 25-75] or n (%).

* Significant difference between NOB and CON *vs.* DOB ( *p* ≤ 0.05);

† Significant difference between NOB *vs* . DOB ( *p* ≤ 0.05);

f Significant difference between DOB *vs* . CON and NOB ( *p* ≤ 0.05).

As for the biochemical profile, fasting plasma glucose, insulin, HOMA-IR, HbA1c, and TNF-α were greater in DOB *vs.* CON ( *p ≤* 0.05, for all outcomes). In addition, DOB exhibited more significant increases than NOB in fasting plasma glucose and HOMA-IR ( *p ≤* 0.02). Any difference between groups was found for lipid profile, ghrelin, and GLP-1 ( *p ≥* 0.06, for all outcomes), but a trend toward lower ghrelin levels in the DOB was noticed.

Results of plasma glucose and gastrointestinal peptides at fasting and 30 and 60 min after standard meal intake are shown in Table 2 . Results from the 3 × 3 ANOVA showed a significant effect of time for ghrelin, GLP-1, and insulin ( *p <* 0.0001), while no significant effect of group and interaction was detected ( *p ≥* 0.08, for all conditions). *Post hoc* analysis showed a substantial reduction in ghrelin between fasting *vs.* 60 min post-meal in all groups ( *p ≤* 0.02). Higher levels of GLP-1 and insulin were detected between fasting *vs.* 30 and 60 min post-meal in all groups ( *p ≤* 0.05), while there was no change between 30 *vs.* 60 min post-meal ( *p ≥* 0.15). There was no difference between groups for ghrelin, GLP-1, and insulin across time ( *p ≥* 0.06).

**Table 2:** Ghrelin, glucagon-like peptide-1, insulin, and glucose levels before and after 30 and 60 min of a standard meal intake according to body adiposity and glucose homeostasis

		Baseline	After		*p* -values		
			30 min	60 min	Time effect	Group effect	Interaction
Ghrelin (pg/mL)							
	CON	81.6 ± 60.6	56.2 ± 45.5	39.1 ± 31.5 *	**<0.0001**	0.08	0.44
	NOB	73.5 ± 35.5	58.8 ± 42.8	39.4 ± 34.2 *			
	DOB	45.3 ± 24.1	32.4 ± 25.5	23.7 ± 15.0 *			
GLP-1 (pM)							
	CON	0.62 [0.43-0.96]	1.95 [0.66-2.10] *	1.06 [0.79-1.74] *	**<0.0001**	0.64	0.97
	NOB	0.59 [0.50-0.73]	1.99 [1.04-2.17] *	1.36 [1.00-2.02] *			
	DOB	0.84 [0.63-0.89]	1.19 [0.84-2.53] *	1.39 [1.19-1.99] *			
Insulin (pg/mL)							
	CON	268.0 ± 280.7	853.8 ± 672.5 *	1030.5 ± 388.2 *	**<0.0001**	0.08	0.36
	NOB	313.2 ± 103.9	1022.7 ± 536.3 *	1466.8 ± 829.5 *			
	DOB	566.7 ± 477.4	1539.6 ± 1200.1 *	1728.9 ± 1109.4 *			
Glucose (mg/dL)							
	CON	95.8 ± 8.1	109.9 ± 11.3 *	109.1 ± 22.7 *	**<0.0001**	**0.003**	**0.01**
	NOB	97.9 ± 7.1	105.9 ± 14.7 *	107.7 ± 10.4 *			
	DOB	140.3 ± 53.6	163.1 ± 67.4 * † f	178.6 ± 65.9 * † f			

CON: normoglycemic eutrophic; NOB: normoglycemic obese; DOB: obese with dysglycemia; GLP-1: glucagon-like peptide-1.

*P* -values of 2-way ANOVA; results expressed as mean ± SD or median [percentiles 25-75].

* Compared to the same group, at fasting ( *P* ≤ 0.05);

† Compared to CON at the same time point ( *P* ≤ 0.05);

f Compared to the NOB at the same time point ( *P* ≤ 0.05).

There was a significant interaction between glucose, with effects for time and group ( *p ≤* 0.01). *Post hoc* analysis detected an increase in plasma glucose at 30 and 60 min compared with fasting in all groups ( *p ≤* 0.05), while no difference was detected between 30 and 60 min ( *p ≥* 0.24). As expected, glucose levels were significantly higher in the DOB compared to the CON after 30 and 60 min of consuming a meal ( *p ≤* 0.05). Similarly, glucose in the DOB was markedly higher than NOB at 30 min and 60 min post-meal ( *p ≤* 0.05). On the other hand, no differences in plasma glucose were observed between CON *vs.* NOB at all time-points ( *p ≥* 0.80).

Significant correlations are as follows: changes in ghrelin were positively correlated with GLP-1 in CON at 30 and 60 min after the meal (r = 0.71, *p =* 0.01 and r = 0.78, *p =* 0.004, respectively). Insulin levels were positively correlated to GLP-1 in CON at fasting (r = 0.75, *p =* 0.007), in DOB at 30 min post-meal (r = 0.53, *p =* 0.04) and in NOB at 60 min post-meal (r = 0,54, *p =* 0.04), as well as to glucose in CON at fasting (r = 0.77, *p =* 0.02) and in NOB at 30 min after the meal (r = 0.78, *p =* 0.007).

## DISCUSSION

The present study investigated the biological behaviors of the active forms of ghrelin and GLP-1 after a standard liquid meal in eutrophic individuals and those with obesity with or without dysglycemia. Our main findings were that a) the circulating levels of active ghrelin were equally reduced in all groups between the fasting *vs.* 60 min post-meal time-points; b) active GLP-1, insulin, and plasma glucose levels increased in all groups between the fasting *vs.* 30 and 60 min post-meal time-points; c) higher increases in plasma glucose were detected in those individuals with obesity and dysglicemia *vs.* controls and normoglycemic individuals with obesity at 30 and 60 min post-meal.

Active ghrelin, an *n* -octanoyl-modified 28-amino-acid-peptide, is an endogenous ligand for the growth hormone secretagogue receptor (GHS-R). Besides inducing growth hormone (GH) secretion, it promotes feeding, increases body weight, controls energy balance, and stimulates gastric acid secretion and gastrointestinal peristalsis ( 16 ). Ghrelin appears to negatively affect energy balance and/or caloric restriction, preventing hypoglycemia and treating clinical conditions for those underweight individuals by stimulating appetite and energy intake. Conversely, ghrelin's inhibition may be somewhat beneficial to control body adiposity through central regulation of food intake ( 17 ).

In contrast to GLP-1, higher ghrelin levels are seen during the fasting state before feeding onset, with subsequent reduction after meal intake ( 18 ); our findings corroborate this result. Glucose load after a meal is associated with a rapid decline in ghrelin level. Since GLP-1 enhances insulin release in response to nutrient ingestion, the inhibition of ghrelin may occur due to a gradual increase in glucose and insulin levels ( 19 ). In addition, other GI hormones such as CCK, GIP, and PYY have possible suppressive effects on ghrelin levels after meal intake, and their synergistic interactions warrant further study ( 20 ).

In this study, with respect to ghrelin levels during fasted or postprandial periods, no differences were detected between individuals with obesity and normal-weight controls, except for a trend toward lower grehlin levels in those with obesity and dysglycemia during the fasting state. This finding is in agreement with previous studies demonstrating that higher adiposity and insulin resistance can influence ghrelin levels ( 8 ). Differences between circadian cycles’ patterns and meal timing, which modulate physiological ghrelin levels, may help explain the absence of significant differences between groups ( 21 ).

In general, postprandial GLP-1 secretion is regulated by a combination of the supply of nutrients in the intestinal lumen, activation of enteric and parasympathetic nervous pathways, and metabolic hormone secretions. Additionally, GLP-1 is rapidly enzymatically metabolized and inactivated by the dipeptidyl peptidase 4 (DPP4) ( 22 ). These previous observations were reproduced in our study; namely, the GLP-1 peak occurred within 30 min post-meal. As expected, insulin levels increased gradually due to GLP-1's incretinomimetic effect, which stimulates insulin release and inhibits glucagon secretion to control postprandial glucose excursions ( 23 ).

Larger increases in postprandial glucose were detected in individuals with obesity and dysglycemia compared with individuals with normal glucose tolerance. This effect may also depend on the macronutrient composition of the meal ingested. In our protocol, the meal was composed of 49% carbohydrates, 35% lipids, and 16% proteins. Meals higher in carbohydrates may effectively suppress ghrelin secretion compared with lipid-rich diets ( 24 ). This effect involves the increase of plasma glucose and insulin levels in response to the ingested meal and renders insulin an inhibitor of ghrelin secretion ( 25 ).

As expected, circulating glucose levels in the DOB group were significantly higher compared with the CON and NOB groups after 30 and 60 min of meal intake. However, during these time-points we did not observe any differences between groups concerning the levels of ghrelin, GLP-1, and insulin. Ghrelin is a peptide hormone produced by enteroendocrine cells located in oxyntic glands of the stomach fundus ( 7 , 26 , 27 ), and its secretion is regulated by energy balance. Secretion is upregulated under conditions of negative energy balance – as in the preprandial state – and downregulated by a positive energy balance, like during the postprandial period ( 1 , 28 , 29 ). During the fasted state, a rise in the levels of total and active (acylated) forms of ghrelin is observed, and a subsequent decline occurs after meal intake ( 18 ). Ghrelin may affect glucose homeostasis by accelerating gastric emptying and inhibiting glucose-induced insulin secretion ( 30 , 31 ). This phenomenon may partly explain the increase in insulin levels according to the decrease of ghrelin after meal intake in all studied groups. According to the literature, ghrelin levels are decreased in patients with obesity and T2DM ( 1 ) compared to eutrophic individuals, due to insulin-inhibitory effects on secretion ( 1 , 7 ). In addition, decreased active ghrelin levels are negatively correlated with abdominal adiposity, hyperinsulinemia, and insulin resistance in T2DM ( 32 ). Moreover, the suppressed ghrelin levels found in patients with obesity and T2DM were followed by reduced postprandial satiety ( 33 , 34 ). In contrast to these findings, with respect to the active ghrelin levels, our study did not demonstrate any difference between CON, NOB, and DOB groups at all time-points.

GLP-1 is a peptide hormone produced in enteroendocrine L cells ( 35 , 36 ) and plays a crucial role in glucose homeostasis. It contributes to meal-related glycemic control by stimulating insulin secretion, inhibiting glucagon secretion, and slowing gastric emptying ( 37 , 38 ). Nevertheless, GLP-1 may also contribute to glycemic control at fasting ( 7 ). GLP-1 is secreted in response to nutrient ingestion and potentiates insulin secretion, the so-called “incretin effect” ( 39 , 40 ). Meal-induced GLP-1 upregulation was reduced with increasing BMI ( 41 – 43 ), especially in the presence of liver steatosis ( 44 ). Faerch and cols. reported that incretin effect of GLP-1 is attenuated in obesity, even in the absence of impaired glucose tolerance or T2DM ( 41 ). Diverging from these data, we did not find significant differences in the active GLP-1 levels between NOB and CON groups before and after standard meal intake. With respect to GLP-1 response to oral glucose in patients with T2DM, data in the literature remain controversial: systematic reviews and meta-analyses reported that GLP-1 secretion is frequently unaltered in T2DM ( 45 ), which is in accordance with our findings. On the other hand, other studies demonstrated that GLP-1 was downregulated ( 43 , 46 ) or even upregulated ( 47 ) in individuals with T2DM.

Limitations of our study include its cross-sectional design, which limits the ability to make cause–effect inferences. Additionally, the narrow BMI range (obesity grades 1 and 2) and the small sample size may have posed barriers to identifying relationships between adiposity and changes in GI peptide levels. Therefore, the contribution of adiposity and glucose homeostasis to regulating ghrelin and GLP-1 levels after meal intake cannot be ruled out entirely. Furthermore, circadian influences must also be considered. Nonetheless, the exclusion of confounding factors would require long-term follow-up studies. Lastly, a more extended profile of the peptide and plasma glucose levels post-meal would have contributed to a more comprehensive data set.

In conclusion, our results showed that the behavior of ghrelin and GLP-1 levels after the standard liquid meal was similar in individuals with different levels of body adiposity and glucose tolerance. Further research is warranted to observe the postprandial time-course of GI hormones and their clinical, metabolic, and behavioral effects on individuals with metabolic diseases.

## References

[1] Tschöp M, Weyer C, Tataranni PA, Devanarayan V, Ravussin E, Heiman ML (2001). Circulating ghrelin levels are decreased in human obesity. Diabetes.

[2] Isaacs D, Prasad-Reddy L, Srivastava SB (2016). Role of glucagon-like peptide 1 receptor agonists in management of obesity. Am J Health Syst Pharm.

[3] Cummings DE, Weigle DS, Frayo RS, Breen PA, Ma MK, Dellinger EP (2002). Plasma ghrelin levels after diet-induced weight loss or gastric bypass surgery. N Engl J Med.

[4] Shah M, Vella A (2014). Effects of GLP-1 on appetite and weight. Rev Endocr Metab Disord.

[5] Ibrahim Abdalla MM (2015). Ghrelin – Physiological Functions and Regulation. Eur Endocrinol.

[6] Tong J, Prigeon RL, Davis HW, Bidlingmaier M, Kahn SE, Cummings DE (2010). Ghrelin suppresses glucose-stimulated insulin secretion and deteriorates glucose tolerance in healthy humans. Diabetes.

[7] Steinert RE, Feinle-Bisset C, Asarian L, Horowitz M, Beglinger C, Geary N (2017). Ghrelin, CCK, GLP-1, and PYY(3-36): Secretory Controls and Physiological Roles in Eating and Glycemia in Health, Obesity, and After RYGB. Physiol Rev.

[8] McLaughlin T, Abbasi F, Lamendola C, Frayo RS, Cummings DE (2004). Plasma ghrelin concentrations are decreased in insulin-resistant obese adults relative to equally obese insulin-sensitive controls. J Clin Endocrinol Metab.

[9] Shah M, Vella A (2014). Effects of GLP-1 on appetite and weight. Rev Endocr Metab Disord.

[10] Holst JJ (2007). The physiology of glucagon-like peptide 1. Physiol Rev.

[11] Jensen MD, Ryan DH, Apovian CM, Ard JD, Comuzzie AG, Donato KA (2014). 2013 AHA/ACC/TOS guideline for the management of overweight and obesity in adults: a report of the American College of Cardiology/American Heart Association Task Force on Practice Guidelines and The Obesity Society. Circulation.

[12] American Diabetes Association (2014). Standards of medical care in diabetes – 2014. Diabetes Care.

[13] Pickering TG, Hall JE, Appel LJ, Falkner BE, Graves J, Hill MN (2005). Recommendations for blood pressure measurement in humans and experimental animals: part 1: blood pressure measurement in humans: a statement for professionals from the Subcommittee of Professional and Public Education of the American Heart Association Council on High Blood Pressure Research. Circulation.

[14] Executive Summary of The Third Report of The National Cholesterol Education Program (NCEP) (2001). Expert Panel on Detection, Evaluation, And Treatment of High Blood Cholesterol In Adults (Adult Treatment Panel III). JAMA.

[15] Matthews DR, Hosker JP, Rudenski AS, Naylor BA, Treacher DF, Turner RC (1985). Homeostasis model assessment: insulin resistance and beta-cell function from fasting plasma glucose and insulin concentrations in man. Diabetologia.

[16] Hosoda H (2022). Effect of Ghrelin on the Cardiovascular System. Biology (Basel).

[17] Cummings DE (2006). Ghrelin and the short- and long-term regulation of appetite and body weight. Physiol Behav.

[18] Cummings DE, Purnell JQ, Frayo RS, Schmidova K, Wisse BE, Weigle DS (2001). A Preprandial Rise in Plasma Ghrelin Levels Suggests a Role in Meal Initiation in Humans. Diabetes.

[19] Pöykkö SM, Kellokoski E, Hörkkö S, Kauma H, Kesäniemi YA, Ukkola O (2003). Low plasma ghrelin is associated with insulin resistance, hypertension, and the prevalence of type 2 diabetes. Diabetes.

[20] Steinert RE, Feinle-Bisset C, Asarian L, Horowitz M, Beglinger C, Geary N (2017). Ghrelin, CCK, GLP-1, and PYY(3-36): Secretory Controls and Physiological Roles in Eating and Glycemia in Health, Obesity, and After RYGB. Physiol Rev.

[21] Spiegel K, Tasali E, Leproult R, Scherberg N, Van Cauter E (2011). Twenty-four-hour profiles of acylated and total ghrelin: relationship with glucose levels and impact of time of day and sleep. J Clin Endocrinol Metab.

[22] Gagnon J, Baggio LL, Drucker DJ, Brubaker PL (2015). Ghrelin Is a Novel Regulator of GLP-1 Secretion. Diabetes.

[23] Page LC, Gastaldelli A, Gray SM, D’Alessio DA, Tong J (2018). Interaction of GLP-1 and Ghrelin on Glucose Tolerance in Healthy Humans. Diabetes.

[24] Foster-Schubert KE, Overduin J, Prudom CE, Liu J, Callahan HS, Gaylinn BD (2008). Acyl and total ghrelin are suppressed strongly by ingested proteins, weakly by lipids, and biphasically by carbohydrates. J Clin Endocrinol Metab.

[25] Erdmann J, Töpsch R, Lippl F, Gussmann P, Schusdziarra V (2004). Postprandial response of plasma ghrelin levels to various test meals in relation to food intake, plasma insulin, and glucose. J Clin Endocrinol Metab.

[26] Kojima M, Hosoda H, Date Y, Nakazato M, Matsuo H, Kangawa K (1999). Ghrelin is a growth-hormone-releasing acylated peptide from stomach. Nature.

[27] Date Y, Kojima M, Hosoda H, Sawaguchi A, Mondal MS, Suganuma T (2000). Ghrelin, a novel growth hormone-releasing acylated peptide, is synthesized in a distinct endocrine cell type in the gastrointestinal tracts of rats and humans. Endocrinology.

[28] Shiiya T, Nakazato M, Mizuta M, Date Y, Mondal MS, Tanaka M (2002). Plasma ghrelin levels in lean and obese humans and the effect of glucose on ghrelin secretion. J Clin Endocrinol Metab.

[29] Castañeda TR, Tong J, Datta R, Culler M, Tschöp MH (2010). Ghrelin in the regulation of body weight and metabolism. Front Neuroendocrinol.

[30] Salehi A, Dornonville de la Cour C, Håkanson R, Lundquist I (2004). Effects of ghrelin on insulin and glucagon secretion: a study of isolated pancreatic islets and intact mice. Regul Pept.

[31] Müller TD, Nogueiras R, Andermann ML, Andrews ZB, Anker SD, Argente J (2015). Ghrelin. Mol Metab.

[32] Katsuki A, Urakawa H, Gabazza EC, Murashima S, Nakatani K, Togashi K (2004). Circulating levels of active ghrelin is associated with abdominal adiposity, hyperinsulinemia and insulin resistance in patients with type 2 diabetes mellitus. Eur J Endocrinol.

[33] Knudsen SH, Karstoft K, Solomon TP (2013). Impaired postprandial fullness in Type 2 diabetic subjects is rescued by acute exercise independently of total and acylated ghrelin. J Appl Physiol (1985).

[34] Knudsen SH, Karstoft K, Solomon TP (2014). Hyperglycemia abolishes meal-induced satiety by a dysregulation of ghrelin and peptide YY3-36 in healthy overweight/obese humans. Am J Physiol Endocrinol Metab.

[35] Gribble FM, Reimann F (2019). Function and mechanisms of enteroendocrine cells and gut hormones in metabolism. Nat Rev Endocrinol.

[36] Hira T, Pinyo J, Hara H (2020). What Is GLP-1 Really Doing in Obesity?. Trends Endocrinol Metab.

[37] Drucker DJ (2006). The biology of incretin hormones. Cell Metab.

[38] Campbell JE, Drucker DJ (2013). Pharmacology, physiology, and mechanisms of incretin hormone action. Cell Metab.

[39] Kieffer TJ, Habener JF (1999). The glucagon-like peptides. Endocr Rev.

[40] D’Alessio D (2016). Is GLP-1 a hormone: Whether and When?. J Diabetes Investig.

[41] Færch K, Torekov SS, Vistisen D, Johansen NB, Witte DR, Jonsson A (2015). GLP-1 Response to Oral Glucose Is Reduced in Prediabetes, Screen-Detected Type 2 Diabetes, and Obesity and Influenced by Sex: The ADDITION-PRO Study. Diabetes.

[42] Ranganath LR, Beety JM, Morgan LM, Wright JW, Howland R, Marks V (1996). Attenuated GLP-1 secretion in obesity: cause or consequence?. Gut.

[43] Muscelli E, Mari A, Casolaro A, Camastra S, Seghieri G, Gastaldelli A (2008). Separate impact of obesity and glucose tolerance on the incretin effect in normal subjects and type 2 diabetic patients. Diabetes.

[44] Matikainen N, Bogl LH, Hakkarainen A, Lundbom J, Lundbom N, Kaprio J (2014). GLP-1 responses are heritable and blunted in acquired obesity with high liver fat and insulin resistance. Diabetes Care.

[45] Nauck MA, Vardarli I, Deacon CF, Holst JJ, Meier JJ (2011). Secretion of glucagon-like peptide-1 (GLP-1) in type 2 diabetes: what is up, what is down?. Diabetologia.

[46] Zhang F, Tang X, Cao H, Lü Q, Li N, Liu Y (2012). Impaired secretion of total glucagon-like peptide-1 in people with impaired fasting glucose combined impaired glucose tolerance. Int J Med Sci.

[47] Alssema M, Rijkelijkhuizen JM, Holst JJ, Teerlink T, Scheffer PG, Eekhoff EM (2013). Preserved GLP-1 and exaggerated GIP secretion in type 2 diabetes and relationships with triglycerides and ALT. Eur J Endocrinol.

